# Miscellanea Miridologica V. Taxonomy and chorology of new or little known taxa of Continental New Guinea and neighboring islands (Insecta, Heteroptera, Miridae)

**DOI:** 10.3897/zookeys.796.20736

**Published:** 2018-11-15

**Authors:** Frédéric Cérot

**Affiliations:** 1 Service Public de Wallonie, DGO3, DEMNA, Av. Maréchal Juin, 23, BE-5030, Gembloux, Belgium, U.E. Service Public de Wallonie Gembloux Belgium

**Keywords:** *
Gressitocoris henryi
*, new species, Miridae, New Guinea, chorology, host-plants

## Abstract

*Gressitocorishenryi* (Deraeocorinae, Deraeocorini) is described as a new species on the basis of the female holotype from Syoubri vill(age), Arfak Mounts, Doberai Peninsula, Papua Barat, Indonesia. Additional data on distribution are provided for 17 species of Cylapinae, Deraeocorinae, Mirinae, Orthotylinae and Phylinae. *Trigonotylustenuis* is cited for the first time from Papua New Guinea.

## Introduction

The recent study of several public and private collections of Miridae (Insecta, Heteroptera) from Iran Jaya or Papua Barat, Papua New Guinea and the Moluccas islands provided several interesting taxonomic and chorological (distributional) data on new or poorly known taxa. When available, data on habitat or assumed host-plants are also given.

## Material and methods

The material used in this work comes mostly from four public and two private collections: the Institut Royal des Sciences Naturelles de Belgique, Brussels (**ISNB**); the Museum National d’Histoire Naturelle, Paris (**MNHN**); the Natural History Museum, London (**NHMUK**); the National Museum of Natural History, Praha (**NMPC**); the collection of D. Telnov in the Erfurt Museum of Natural History, Erfurt (ex **DTPC**, **NME**); and the private collection of J. Gorczyca (**JGKP**).

In the descriptions, measurements are given in millimeters (mm). The photos showing morphological details were taken with a Nikon DXM1200 digital camera.

## Results

### Cylapinae Kirkaldy, 1903

#### Fulviini Uhler, 1886

##### Cylapofulvius punctatus

Taxon classificationAnimaliaHeteropteraMiridae

Poppius, 1909

###### Material examined.

Indonesia: 1♂, Papua Barat, Doberai Peninsula, Arfak Mounts, Syoubri vill(age) (coordinates provided on the label: 1°07'16"S, 133°54'34"E), 1570–2100 m, primary lower mountain rainforest, 11–12.ix.2015, Telnov D. leg. (FC n° 7580) (ex DTPC, NME).

###### Distribution.

Described from Papua New Guinea and also known from Papua Barat and Solomon Islands (Chérot et al. 2017).

##### Fulvius subnitens

Taxon classificationAnimaliaHeteropteraMiridae

Poppius, 1909

###### Material examined.

Papua New Guinea: 2♀♀, Madang Province, Nagada Binatang Research Center (coordinates provided on the label: 05°09'23"S, 145°47'41"E), 20 m, 20.v.2011, Votýpka J. & Lukeš J. leg. (FC n° 6420) (NMPC).

###### Distribution.

Described at least in part from Papua New Guinea. Widely distributed from Africa (Tanzania, Togo) and the Seychelles to Taiwan and Pacific Islands and from Malaysia to Papua New Guinea (Chérot et al. 2017); recently introduced but not established in several European countries.

##### Fulvius variegatus

Taxon classificationAnimaliaHeteropteraMiridae

Poppius, 1909

###### Material examined.

Papua New Guinea: 1?, Madang Province, Baitbag (coordinates provided on the label: 05°08'46"S, 145°46'36"E), 40 m, 17.v.2011, dissected for parasites, dissection number 716, negative, Votýpka J. & Lukeš J. leg. (FC n° 6437) (NMPC).

###### Distribution.

Described from Papua New Guinea and widely distributed in Pacific Islands (Chérot et al. 2017).

### Deraeocorinae Douglas & Scott, 1865

#### Deraeocorini Douglas & Scott, 1865

##### Deraeocoris finisterrensis

Taxon classificationAnimaliaHeteropteraMiridae

Carvalho, 1985

###### Material examined.

Indonesia: 1♀, Papua Barat, Doberai Peninsula, Arfak Mounts, Syoubri vill(age) (coordinates provided on the label: 1°06'40"S, 133°54'36"E), 1510 m, edge of secondary lower mountain rainforest, at white light, 11–12.ix.2015, Telnov D. leg. (FC n° 7565) (ex DTPC, NME); 8♀♀, Papua Barat, Doberai Peninsula, Arfak Mounts, Anggi Gigi Lake S(outh), upper vill(age) (coordinates provided on the label: 1°18'0.5"S, 133°54'24"E), 2200 m, edge of primary mid mountain rainforest, 09–11.ix.2015, Telnov D. leg. (FC n°s 7560–7564, 7567, 7569, 7571) (ex DTPC, NME); 1♀, 1?, Papua Barat, Doberai Peninsula, Arfak Mounts, Anggi Gigi Lake S(outh), upper vill(age) and surroundings (coordinates provided on the label: 1°18'10"S, 133°54'0.3"E), 1985 m, primary mid montain rainforest, at white light, 08–09.xi.2015, Telnov D. leg. (FC n°s 7562, 7562bis) (ex DTPC, NME).

###### Distribution.

Endemic to New Guinea (Schuh 2002–2013).

##### Gressitocoris henryi sp. n.

Taxon classificationAnimaliaHeteropteraMiridae

http://zoobank.org/2FFAE8FA-E8C0-448D-9BCA-5142027591A5

###### Material examined.

Indonesia: Holotype ♀, Papua Barat, Doberai Peninsula, Arfak Mounts, Syoubri vill(age) (coordinates provided on the label: 1°06'40"S, 133°54'36"E), 1510 m, edge of secondary lower mountain rainforest, at white light, 12–13.ix.2015, Telnov D. leg. (FC n° 7565). Holotype deposited in DTPC, NME.

###### Description.

Female: *Measurements (mm)*: Total length (dorsal view): 7.00, maximal width of hemelytra: 2.95, width of head across eyes (“diatone”): 1.25, width of vertex: 0.45, length of antennal segments: I: 0.70, II: 1.68, III: 1.10, IV: 0.75, medial pronotal length (pronotal collar included): 1.45, posterior pronotal width (between humeral angles): 2.40, lateral length of pronotum (between anterior and humeral angles): 1.25, length of scutellum: 1.20, width of scutellum: 1.30, length of cuneus: 1.08, width of cuneus: 0.60 (0.75 with paracuneus).

###### External morphology and coloration.

**Figures 1–10.**
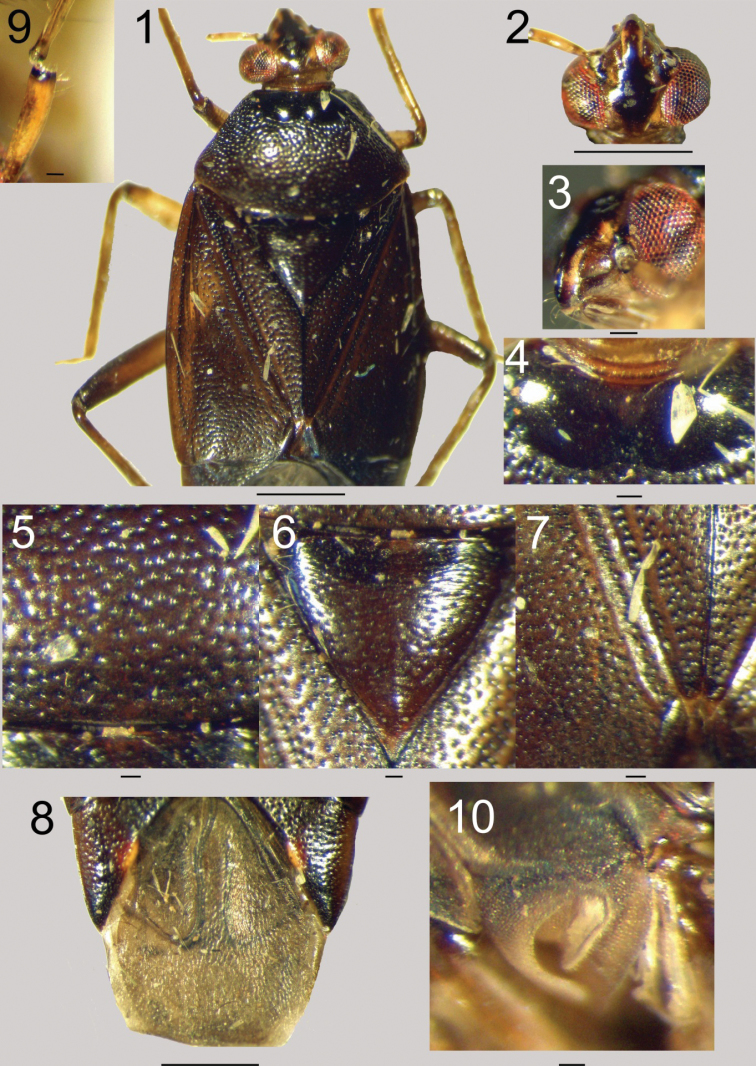
*Gressitocorishenryi* sp. n. female holotype. **1** Habitus in dorsal view **2** Head in dorsal view **3** Head in lateral view **4** Pronotal callosities **5** Pronotal disk in dorsal view **6** Scutellum in dorsal view **7** Endocorium and apex of clavi in dorsal view **8** Membrane and cunei in dorsal view **9** Left antenna: first antennal segment and base of the second **10** Evaporative area. Scale bar = 0.1 mm (except in Figs 1, 2 and 8, scale = 1.0 mm).

Dorsally glabrous on pronotum, scutellum and hemelytra. Head (Figs 1–3): Elongate, smooth, slightly declivous in dorsal view. Clypeus medially black, laterally yellowish (Figure 2). Mandibulary and maxillary plates dark brown to black (Figure 3). Frons smooth, shining black. Vertex slightly carinate, carina brown, surface of vertex narrowly and shallowly punctate posteriorly, smooth anteriorly, dark brown to shining black medially, with two small yellowish areas near inner margins of eyes, prolonged on frons. Eyes reddish with several black patches medially (Figure 2), occupying head height in lateral view (Figure 3). First antennal segment thickened sub-basally, after small concavity, slightly longer than vertex width, yellowish brown, with apical black ring (Figure 9), apparently devoid of erect setae. Second segment narrower, significantly longer, yellowish brown, darker apically, with several erect setae obviously longer than width of segment. Third and fourth segments dark brown to black, with same erect pilosity. Labium reaching metacoxae, yellowish brown. Pronotum (Figs 1, 4–5): Pronotal collar (Figure 4) very short, brown, almost smooth, with very narrow and shallow punctation. Pronotal callosities (Figure 4) rounded, medially separated and separated from pronotal lateral margins, shining black, smooth. Pronotal lateral margins slightly concave to sigmoid medially, carinate, carina yellowish, easily visible in lateral view. Pronotal posterior margin (Figure 1) convex but medially almost straight and laterally, near humeral angles, slightly concave. Humeral angles rounded. Pronotal disk widely and deeply punctate (Figure 5), punctation dense, black, surface of disk dark brown. Mesoscutum covered (Figure 6). Scutellum (Figure 6) slightly swollen, reddish brown to dark brown, more narrowly punctate. Clavus and corium (Figure 7), including embolium, widely and deeply punctate, punctation black, surface of hemelytra evenly dark brown. Cuneus (Figure 8) dark brown bearing inner reddish sub-basal spot with wide whitish inner margin. Membrane (Figure 8) slightly declivous, greyish, veins thick, blackish to greenish, larger cell curved inward submedially. Coxae yellow. Pro- and mesofemora yellowish, darker apically. Metafemora dark brown to black. Metatibiae yellowish brown, as tarsi. Claws reddish. Pilosity of legs elongate, stiff, about as long as tibial spine. Propleura almost black, narrowly and shallowly punctate. Meso- and metapleura dull, blackish with yellowish areas. Abdomen dark brown, with elongate white setae.

###### Genital structures.

Not dissected to preserve the holotype.

Male unknown.

###### Etymology.

I am pleased to dedicate this new species to Dr T. J. Henry (United States National Museum of Natural History, Washington D.C, United States of America) in recognition of his major contributions to Heteroptera taxonomy, particularly to the classification and phylogeny of Berytidae and Lygaeoidea, but also to the study of several difficult plant bug genera such *Ceratocapsus* Reuter, 1876, *Hyalochloria* Reuter, 1907, *Neurocolpus* Reuter, 1876 and *Ranzovius* Distant, 1893.

###### Discussion.

Through the courtesy of Dr T. J. Henry, I was able to compare the new species to the dorsal and lateral views of a paratype of *G.sedlaceki* Carvalho, 1985, the type species of *Gressitocoris* and, until now the only species of the genus. The female holotype of the new species concords with Carvalho’s (1985) original description of *Gressitocoris* in a majority of character states. The antennal segments are covered by dense pilosity with some sparse, erect setae longer than width of the segment, the second antennal segment is slightly thickened apically, the posterior margin of pronotal disk is rounded but slightly concave laterally near humeral angles, the lateral margins are carinate, the pronotal disk and hemelytra (including wide embolium) are widely and deeply punctate, the scutellum is more narrowly punctate, the vein of larger areolar cell of the membrane is thick, expanded posteriorly, curved inward submedially and a reddish sub-basal spot with wide whitish inner margin is present on inner part of cuneus.

A very narrow and shallow punctation is apparently present on the pronotal collar of both species (contra Carvalho 1985).

*Gressitocorishenryi* sp. n. differs from *G.sedlaceki* Carvalho, 1985 by the length of the third antennal segment shorter than the length of the second antennal segment (versus slightly longer in *G.sedlaceki*), the eyes less wide, the covered mesoscutum and the darker dorsal coloration, particularly the medial black stripe of clypeus, the medial black patch of frons and vertex (both absent in *G.sedlaceki*), the almost even dark brown to black pronotum (yellowish brown lateral areas and posterior margin absent), the reddish brown scutellum (yellowish lateral stripes absent), the almost even dark brown hemelytra, and absence of an elongate yellowish stripe lining the clavo-corial suture.

As pointed out by T. Yasunaga (in litt. 2017-08-22), the validity of the genus *Gressitocoris* Carvalho, 1985 should be analyzed and compared with the large genus *Deraeocoris* Kirschbaum, 1856, whose monophyly remains to be established. However, *Gressitocorishenryi* sp. n. differs in habitus from all Papuan species of *Deraeocoris* described or redescribed by Carvalho (1985).

###### Distribution.

Indonesia, Papua Barat, Doberai Peninsula. Type locality: Syoubri vill(age) (1°06'40"S, 133°54'36"E).

#### Saturniomirini Carvalho, 1952

##### Imogen bicolor

Taxon classificationAnimaliaHeteropteraMiridae

(Poppius, 1912a)

###### Material examined.

Indonesia: 1♂, North Moluccas, Central Halmahera, creek N(orth)-E(ast) of Kobe vill(age), creek side (coordinates provided on the label: 0°28'41"N, 127°53'53"E), 10 m, 07.vii.2013, Telnov D. leg. (FC n° 7559) (ex DTPC, NME).

###### Distribution.

According to Chérot et al. (2017), this species was described from a small series of males from Ighibirei, Central Province, Papua New Guinea and is known from continental Papua Barat, Indonesia, and from Papua New Guinea. This is apparently the first citation from the North Moluccas.

### Mirinae Hahn, 1833

#### Mirini Hahn, 1833

##### Moroca giluwensis

Taxon classificationAnimaliaHeteropteraMiridae

Carvalho, 1986

###### Material examined.

Papua New Guinea: 1♀, Eastern Highlands, Goroka (coordinates provided on the label: 6°4'44"S, 145°22'56"E), 1600 m, 13.v.2011, dissected for parasites, dissection number 627, negative, Votýpka J. & Lukeš J. leg. (FC n° 6420) (NMPC); 1?, M(oun)t Gahavisuka Provincial Park (coordinates provided on the label: 6°2'2"S, 145°25'28"E), 2000 m, 11.v.2011, dissected for parasites, dissection number 597, negative, Votýpka J. & Lukeš J. leg. (FC n° 6421) (NMPC).

###### Distribution.

Endemic to New Guinea (Schuh 2002–2013).

##### Moroca lineaticolle

Taxon classificationAnimaliaHeteropteraMiridae

Poppius, 1912b

###### Material examined.

Indonesia: 1♀, Papua Barat, Doberai Peninsula, Arfak Mounts, Anggi Gigi Lake S(outh), upper vill(age) (coordinates provided on the label: 1°18'0.5"S, 133°54'24"E), 2200 m, edge of primary mid mountain rainforest, 09–11.ix.2015, Telnov D. leg. (FC n° 7574) (ex DTPC, NME); 1♀, Papua Barat, Doberai Peninsula, Arfak Mounts, Syoubri vill(age) (coordinates provided on the label: 1°06'40"S, 133°54'36"E), 1510 m, edge of secondary lower mountain rainforest, at white light, 12–13.ix.2015, Telnov D. leg. (FC n° 7575) (ex DTPC, NME).

###### Distribution.

Endemic to New Guinea (Schuh 2002–2013).

##### Moroca verticillata

Taxon classificationAnimaliaHeteropteraMiridae

Carvalho, 1986

###### Material examined.

Indonesia: 1♂, 1♀, Papua Barat, Doberai Peninsula, Arfak Mounts, Syoubri vill(age) (coordinates provided on the label: 1°06'40"S, 133°54'36"E), 1510 m, edge of secondary lower mountain rainforest, at MV light, 12–13.ix.2015, Telnov D. leg. (FC n°s 7572–7573) (ex DTPC, NME).

###### Distribution.

Endemic to New Guinea (Schuh 2002–2013).

##### Peltidopeplus annulipes

Taxon classificationAnimaliaHeteropteraMiridae

Poppius, 1912b

###### Material examined.

Indonesia: 1♀, Papua Barat, Doberai Peninsula, Arfak Mounts, Anggi Gigi Lake S(outh), upper vill(age) and surroundings (coordinates provided on the label: 1°18'0.5"S, 133°54'24"E), 2200 m, primary mid mountain rainforest, at white light, 10–11.ix.2015, Telnov D. leg. (FC n° 7551) (ex DTPC, NME); 1♀, Papua Barat, Doberai Peninsula, Arfak Mounts, Anggi Gigi Lake S(outh), upper vill(age) and surroundings (coordinates provided on the label: 1°18'10"S, 133°54'0,3"E), 1985 m, primary mid mountain rainforest, at white light, 08–09.xi.2015, Telnov D. leg. (FC n° 7552) (ex DTPC, NME).

###### Distribution.

Endemic to New Guinea (Chérot and Pauwels 2000; Schuh 2002–2013).

##### Prolygus alboscutellata

Taxon classificationAnimaliaHeteropteraMiridae

Carvalho, 1987a

###### Material examined.

Papua New Guinea: 1♂, 1♀, 1?, Eastern Highlands, Kegsugl, under M(oun)t Wilhelm (coordinates provided on the label: 05°49'52"S, 145°05'10"E), 2780 m, 13.v.2012, dissected for parasites, dissection numbers 623–625, negative, Votýpka J. & Lukeš J. leg. (FC n° 6420) (NMPC).

###### Distribution.

Endemic to New Guinea (Schuh 2002–2013).

##### Prolygus papuanus

Taxon classificationAnimaliaHeteropteraMiridae

(Poppius, 1914)

###### Material examined.

Indonesia: 11♂♂, 13♀♀, 2??, Papua Barat, Doberai Peninsula, Arfak Mounts, Anggi Gigi Lake S(outh), upper vill(age) (coordinates provided on the label: 1°18'0.5"S, 133°54'24"E), 2200 m, edge of primary mid mountain rainforest, at white light, 09–11.ix.2015, Telnov D. leg. (FC n°s 7595–7621) (ex DTPC, NME); 34♂♂, 20♀♀, 5??, Papua Barat, Doberai Peninsula, Arfak Mounts, Syoubri vill(age) (coordinates provided on the label: 1°06'40"S, 133°54'36"E), 1610 m, edge of secondary lower mountain rainforest, 12–13.ix.2015, Telnov D. leg. (FC n° 7622–7681) (ex DTPC, NME).

###### Distribution.

Endemic to New Guinea (Schuh 2002–2013).

###### Remark.

The genital structures of the male FC n° 7771 conform to Carvalho’s (1987a: 148, figs 44–46) drawings.

##### Tinginotum knolwesi

Taxon classificationAnimaliaHeteropteraMiridae

(Kirkaldy, 1908)

###### Material examined.

Indonesia: 2♂♂, 2♀♀, Papua Barat, Paniai (? Regency), Sinak (no coordinates on the label, approximate coordinates available via Google Earth for Paniai Regency: 3°47'S, 136°21'E), 14–17.xii.1995, Riedel A. leg. (FC n°s 7335–7338) (JGKP).

###### Distribution.

Widely distributed in New Guinea and Pacific Islands (Schuh 2002–2013, Chérot et al. 2017).

##### Tinginotum rubrovenosus

Taxon classificationAnimaliaHeteropteraMiridae

Carvalho, 1987b

###### Material examined.

Indonesia: 1♂, Papua Barat, Doberai Peninsula, Arfak Mounts, Anggi Gigi Lake S(outh), upper vill(age) (coordinates provided on the label: 1°18'0.5"S, 133°54'24"E), 2200 m, edge of primary mid mountain rainforest, at white light, 09–11.ix.2015, Telnov D. leg. (FC n° 7576) (ex DTPC, NME); 2♂♂, 1♀(?), Papua Barat, Doberai Peninsula, Arfak Mounts, Syoubri vill(age) (coordinates provided on the label: 1°06'40"S, 133°54'36"E), 1510 m, edge of secondary lower mountain rainforest, 12–13.ix.2015, Telnov D. leg. (FC n° 7577–7579) (ex DTPC, NME).

###### Distribution.

Endemic to New Guinea (Schuh 2002–2013).

##### Warrissia huonensis

Taxon classificationAnimaliaHeteropteraMiridae

(Poppius, 1914a)

###### Material examined.

Indonesia: 1?, Papua Barat, Doberai Peninsula, Arfak Mounts, Syoubri vill(age) (coordinates provided on the label: 1°07'16"S, 133°54'34"E), 1570–2100 m, primary lower mountain rainforest, 11–12.ix.2015, Telnov D. leg. (FC n° 7558) (ex DTPC, NME); 2♀♀, 3??: Papua Barat, Doberai Peninsula, Arfak Mounts, Syoubri vill(age) (coordinates provided on the label: 1°06'40"S, 133°54'36"E), 1510 m, edge of secondary lower mountain rainforest, MV light, 12–13.ix.2015, Telnov D. leg. (FC n°s 7553–7557) (ex DTPC, NME); Papua New Guinea: 1♂, Madang Province, Nagada Harbour (no coordinates on the label, approximate coordinates available via Google Earth: Latitude: -5.16667 Longitude: 145.81667), 18.iii.1990, Lansbury I. leg. (FC n° 7333) (JGKP); 1♀, Madang Province, Nagada Harbour (no coordinate on the label, approximate coordinates available via Google Earth: Latitude: -5.16667 Longitude: 145.81667), 16.v.1992, Lansbury I. leg. (FC n° 7334) (JGKP).

###### Distribution.

Widespread in Southeast Asia and New Guinea (Yasunaga et al. 2002, Schuh 2002–2013, Chérot et al. 2017).

#### Stenodemini China, 1943

##### Trigonotylus tenuis

Taxon classificationAnimaliaHeteropteraMiridae

Reuter, 1893

###### Material examined.

Papua New Guinea: 2♂♂, Madang Province, Nagada Harbour (no coordinates on the label, approximate coordinates available via Google Earth: Latitude: -5.16667 Longitude: 145.81667), 18.iii.1990, Lansbury I. leg. (FC n°s 7340, 7342) (JGKP); 1♂, 1♀, Madang Province, Nagada Harbour (no coordinates on the label, approximate coordinates available via Google Earth: Latitude: -5.16667 Longitude: 145.81667), 16.v.1992, Lansbury I. leg. (FC n°s 7339, 7341) (JGKP).

###### Distribution.

World distribution widespread (Schuh 2002–2013), but apparently not previously recorded from Papua New Guinea.

### Orthotylinae Van Duzee, 1916

#### Austromirini Carvalho, 1976

##### Irianocoris italae

Taxon classificationAnimaliaHeteropteraMiridae

Carvalho, 1971

###### Material examined.

Papua New Guinea: 1♂, Madang Province, Baiteta Forest (no coordinates provided on label; according to Chérot et al. 2017, Baiteta lies at the following coordinates: 05°01'S, 145°45'E), 11.v.1995, O. Missa leg., host unknown, fogging (FC n°4083) (ISNB); 2♂♂, 4♀♀, same locality, 25.v.1995, O. Missa leg., *Planchonellathysoidis* or *Dysoxylumarnoldicum* (Sapotaceae + Melicaceae), fogging (FC n°s 4084–4089) (ISNB); 3♀♀, same locality, 08.vi.1995, O. Missa leg., *Hapholobus* sp. (Burseraceae), fogging (FC n°s 4090–4092) (ISNB); 2♂♂, same locality, 09.vi.1995, O. Missa leg., *Sarcocephalus* sp. (Rubiaceae), fogging (FC n°s 4093–4094) (ISNB); 1♂, 2♀♀, 1?, same locality, 14.vi.1995, O. Missa leg., *Chisochetonceramicus* (Melicaceae), fogging (FC n°s 4095–4097, 4173) (ISNB); 6♂♂, 2??, same locality, 14.vii.1995, O. Missa leg., *Neonauclea* sp. (Rubiaceae), fogging (FC n°s 4098–4103, 4105, 4109) (ISNB); 3♂♂, same locality, 17.vii.1995, O. Missa leg., *Chisochetonwenlandia* (Melicaceae), fogging (FC n°s 4106–4108) (ISNB); 3♂♂, 5♀♀, 3?, same locality, 1995, O. Missa leg., *Celtislatifolia* (Ulmaceae) or *Planchonella* sp. (Sapotaceae), fogging (FC n°s 4110–4120) (ISNB); 6♀♀, 1?, same locality, 1995, O. Missa leg., *Chisochetonceramicus* (Melicaceae), fogging (FC n°s 4121–4127) (ISNB); 1♂, same locality, 10.iv.1996, O. Missa leg., *Litesiairianensis* (Liliaceae), light trap (FC n°4128) (ISNB); 1♂, 2♀♀, same locality, 17.iv.1996, O. Missa leg., host unknown, fogging (FC n°s 4129–4131) (ISNB); 1♂, same locality, ?.iv.1996, O. Missa leg., host unknown, light trap (FC n°4102b) (ISNB); 2♀♀, same locality, 01.v.1996, O. Missa leg., host unknown, fogging (FC n°s 4132–4133) (ISNB); 2♂♂, 2♀♀, same locality, 07.vi.1996, O. Missa leg., host unknown, fogging (FC n°s 4134–4137) (ISNB); 1?, same locality, 04.vii.1996, O. Missa leg., on *Sloaneasogeriensis* (Elaeocarpaceae), fogging (FC n° 4138) (ISNB); 1♂, same locality, 10.vii.1996, O. Missa leg., *Celtisphilippinensis* (Ulmaceae) or *Polyorthia* sp. (Annonaceae), light trap (FC n°4139) (ISNB); 1♂, 1♀, 1?, same locality, 25.vii.1996, O. Missa leg., host unknown, fogging (FC n°s 4140–4142) (ISNB).

###### Distribution.

Endemic to New Guinea. Described by Carvalho (1971) from Maffin Bay, Dutch New Guinea, the species was recently cited for the first time from Papua New Guinea (Cassis et al. 2012).

###### Remark.

The genus *Irianocoris* was transferred from Orthotylini to Austromirini by Cassis, Cheng and Tatarnic (2012).

### Phylinae Douglas & Scott, 1865

#### Hallodapini Van Duzee, 1916 sensu Schuh & Menard 2013

##### Linacoris viridescens

Taxon classificationAnimaliaHeteropteraMiridae

Carvalho, 1983

###### Material examined.

Papua New Guinea: 4♂♂, 2♀♀, 2??, Madang Province, Nagada Binatang Research Center (coordinates provided on the label: 05°09'23"S, 145°47'41"E), 20 m, 20.v.2011, two specimens dissected for parasites, dissection numbers 892–893, negative, Votýpka J. & Lukeš J. leg. (FC n°s 6450–6453, 6455–6458) (NMPC); 2♂♂, 1♀, Madang Province, Ohu (coordinates provided on the label: 05°08'46"S, 145°46'36"E), 06.v.2011, dissected for parasites, dissection numbers 205–207, negative, Votýpka J. & Lukeš J. leg. (FC n°s 6454, 6459–6460) (NMPC).

###### Distribution.

Described by Carvalho (1983) from Papua New Guinea and Iran Jaya. Additional species of the genus *Linacoris* Carvalho, 1983 remain to be described in Southeast Asia (Schuh and Menard 2013).

###### Remark.

The genus *Linacoris* was recently transferred from Orthotylinae, Orthotylini to Phylinae, Hallodapini by Schuh and Menard (2013) on the basis of phylogenetic analyses by Menard et al. (2013).

## Supplementary Material

XML Treatment for Cylapofulvius punctatus

XML Treatment for Fulvius subnitens

XML Treatment for Fulvius variegatus

XML Treatment for Deraeocoris finisterrensis

XML Treatment for Gressitocoris henryi

XML Treatment for Imogen bicolor

XML Treatment for Moroca giluwensis

XML Treatment for Moroca lineaticolle

XML Treatment for Moroca verticillata

XML Treatment for Peltidopeplus annulipes

XML Treatment for Prolygus alboscutellata

XML Treatment for Prolygus papuanus

XML Treatment for Tinginotum knolwesi

XML Treatment for Tinginotum rubrovenosus

XML Treatment for Warrissia huonensis

XML Treatment for Trigonotylus tenuis

XML Treatment for Irianocoris italae

XML Treatment for Linacoris viridescens

## References

[B1] Carvalho JCM (1952) On the major classification of the Miridae (Hemiptera, Heteroptera) (with keys to subfamilies and tribes and a catalogue of the world genera). Anais da Academia brasileira de Ciencias 24: 31–110.

[B2] Carvalho JCM (1971) Sôbre uma curiosa espécies nova de Mirídeo da Nova Guiné. Revista Brasileira de Biologia 31(1): 15–16.

[B3] Carvalho JCM (1976) Analecta miridologica: concerning changes of taxonomic positions of some genera and species. Revista Brasileira de Biologia 36(1): 49–59.

[B4] Carvalho JCM (1983) A new genus and four new species from Oceania (Hemiptera). Revista Brasileira de Biologia 43(4): 401–408.

[B5] Carvalho JCM (1985) On some species of the tribe Deraeocorini Douglas & Scott from Papua New Guinea (Hemiptera). Revista Brasileira de Biologia 45(4): 447–470.

[B6] Carvalho JCM (1986) On the genus *Moroca* Poppius from Papua New Guinea with descriptions of sixteen new species (Hemiptera, Miridae). Revista Brasileira de Biologia 46(4): 757–776.

[B7] Carvalho JCM (1987a) *Prolygus* n.gen. with descriptions of new species and redescription of known ones from Papua New Guinea (Hemiptera, Miridae). Revista Brasileira de Biologia 47 (1/2): 137–153.

[B8] Carvalho JCM (1987b) The genera *Tinginotopsis* Poppius and *Tinginotum* Kirkaldy from Papua New Guinea (Hemiptera, Miridae). Revista Brasileira de Biologia 47(1/2): 165–176.

[B9] Cassis G Cheng M Tatarnic NJ (2012) Systematics of the Plantbug Genus *Irianocoris* Carvalho (Insecta: Heteroptera: Miridae: Orthotylinae: Austromirini). Entomologica Americana 118(1): 157–176. 10.1664/12-RA-039.1

[B10] Chérot F Gorczyca J Schwartz MD Demol T (2017) The Bryocorinae, Cylapinae, Deraeocorinae and Mirinae (Insecta: Heteroptera: Miridae) from Baiteta Forest, Papua New Guinea, with a discussion of their feeding habits and a list of species of the country. In: Telnov D Barclay MVL Pauwels OSG (Eds) Biodiversity, Biogeography and Nature Conservation in Wallacea and New Guinea Vol.III. The Entomological Society of Latvia, Riga, 55–139. [pls 7–18]

[B11] Chérot F Pauwels OSG (2000) Révision du genre *Peltidopeplus* Poppius, 1912, avec description d’une espèce nouvelle de Papouasie et d’un genre nouveau d’Australie (Insecta, Heteroptera, Miridae: Mirini). Zoosystema 22(1): 121–137.

[B12] China WE (1943) Part 8. The generic names of the British HemipteraHeteroptera, with a check list of British Species. Royal Entomological Society, London, 131 pp.

[B13] Distant WL (1893) Insecta Rhynchota Hemiptera-Heteroptera. Biologia Centrali Americana, Suppl., Porter, London, 1, i-xx, 329–462.

[B14] Douglas JW Scott J (1865) The British-Hemiptera. Vol. 1. Hemiptera-Heteroptera. The Ray Society, London, 627 pp. [21 plates]

[B15] Hahn CW (1833) Die wanzenartigen Insecten. 1. C. H. Zeh, Nurnberg, 117 pp.

[B16] Kirkaldy GW (1903) Einige neue und wenig bekannte Rhynchoten. Wiener Entomologische Zeitung 22: 13–16.

[B17] Kirkaldy GW (1908) A catalogue of the Hemiptera of the Fiji. Proceedings of the Linnean Society of New South Wales 33(1907): 345–391.

[B18] Kirschbaum CL (1856) Rhynchotographische Beiträge. Jahrbuch des Vereins fur Naturkunde im Herzgothum Nassau 10(1855): 163–348.

[B19] Menard KL Schuh RT Woolley JB (2013) Total-evidence phylogenetic analysis and reclassification of the Phylinae (Insecta: Heteroptera: Miridae), with the recognition of new tribes and subtribes and a redefinition of Phylini. Cladistics 2013: 1–37. 10.1111/cla.12052 34788969

[B20] Poppius B (1909) Zur Kenntnis der Miriden-Unterfamilie Cylapina Reuter. Acta Societatis Scientiarum Fennicae 37(4) 17: 1–46.

[B21] Poppius B (1912a) Über die Gattung *Araspus* Distant (Hemiptera, Miridae). Wiener Entomologische Zeitung 31(6–7): 227–232.

[B22] Poppius B (1912b) Neue oder weinig bekannte Capsarien-Gattungen und Arten. Annales Musei Nationalis Hungarici 10: 415–441.

[B23] Poppius B (1914a) Zur Kenntnis der Indo-Australischen *Lygus*-Arten. Annales Musei Nationalis Hungarici 12: 337–398.

[B24] Reuter OM (1876) Capsinae ex America Boreali in Museo Holmiensi asservatae, descriptae. Öfversigt af Kongliga Vetenskapsakademiens Förhandlingar 32(9) (1875): 59–92.

[B25] Reuter OM (1893) [Espèce nouvelles de Miridae]. In: Bergroth E (Ed.) Mission scientifique de M. Ch. Alluaud aux Iles Séchelles (mars, avril, mai 1892).Revue d'Entomologie 12: 197–209.

[B26] Reuter OM (1907) Capsidae novae in insula Jamaica mense Aprilis 1906 a D. E. P. Van Duzee collectae. Öfversigt af Finska Vetenskapssocietetens Förhandlingar 49(5): 1–27.

[B27] Schuh RT (1995) Plant bugs of the world (Insecta: Heteroptera: Miridae). Systematic Catalog, Distributions, Host List, and Bibliography. Entomological Society of New York, 1329 pp.

[B28] Schuh RT (2002–2013) On-line Systematic Catalog of Plant Bugs (Insecta: Heteroptera: Miridae). http://research.amnh.org/pbi/catalog/ [last access: 21/04/2016]

[B29] Schuh RT Menard KL (2013) A revised classification of the Phylinae (Insecta: Heteroptera: Miridae): Arguments for the placement of genera. American Museum Novitates 3785: 1–72. 10.1206/3785.2

[B30] Uhler PR (1886) Check-list of the HemipteraHeteroptera of North America. Brooklyn Entomological Society, 1–32. [not seen]

[B31] Van Duzee EP (1916) Synoptical keys to the genera of North American Miridae. University of California Publications in Entomology, Technical Bulletin 1: 199–216.

[B32] Yasunaga T Schwartz MD Chérot F (2002) New genera, species, synonymies and combinations in the “*Lygus* Complex” from Japan, with discussion on *Peltidolygus* Poppius and *Warrisia* Carvalho (Heteroptera: Miridae: Mirinae). American Museum Novitates 3378: 1–26. 10.1206/0003-0082(2002)378%3C0001:NGSSAC%3E2.0.CO;2

